# Supplementary material for: Autistic traits foster effective curiosity-driven exploration
Source: PLoS Comput Biol. 2024 Oct 31;20(10):e1012453. doi: 10.1371/journal.pcbi.1012453 (PMC11527316; doi:10.1371/journal.pcbi.1012453)
Supplement: S1 Text — Fig A. The preprocessing pipeline. Fig B. Participants’ predictions. Fig C. Model simulations. Fig D. Parameters recovery. Fig E. Model comparison. Fig F. Comparing behavioral data to the predictions of the logistic model. Table A. Relation between leave-stay decisions and other-reports. Table B. Relation between leave-stay decisions and self-reports. Appendix A. Task-related questionnaire. Appendix B. Supplementary analyses. (DOCX) [file pcbi.1012453.s001.docx]

### Supporting Information

### Autistic traits foster effective curiosity-driven exploration

**Text S1. Supplementary tables, figures, and analyses.**

**Appendix A: Task-related questionnaire**

|  | Strongly disagree | Partly disagree | Neutral | Partly agree | Strongly agree |
| --- | --- | --- | --- | --- | --- |
| I didn't like the task |  |  |  |  |  |
| I found the instructions clear and knew what I had to and could do |  |  |  |  |  |
| Not enough time was spent in each environment. |  |  |  |  |  |
| I liked the task |  |  |  |  |  |
| The task was over too quickly. |  |  |  |  |  |
| I found the instructions unclear and didn't really know what to do. |  |  |  |  |  |
| My choices while playing were random and based on nothing. |  |  |  |  |  |
| Too much time was spent in each environment. |  |  |  |  |  |
| The task took too long |  |  |  |  |  |
| My choices while playing were based on a (self-chosen) strategy. |  |  |  |  |  |

**
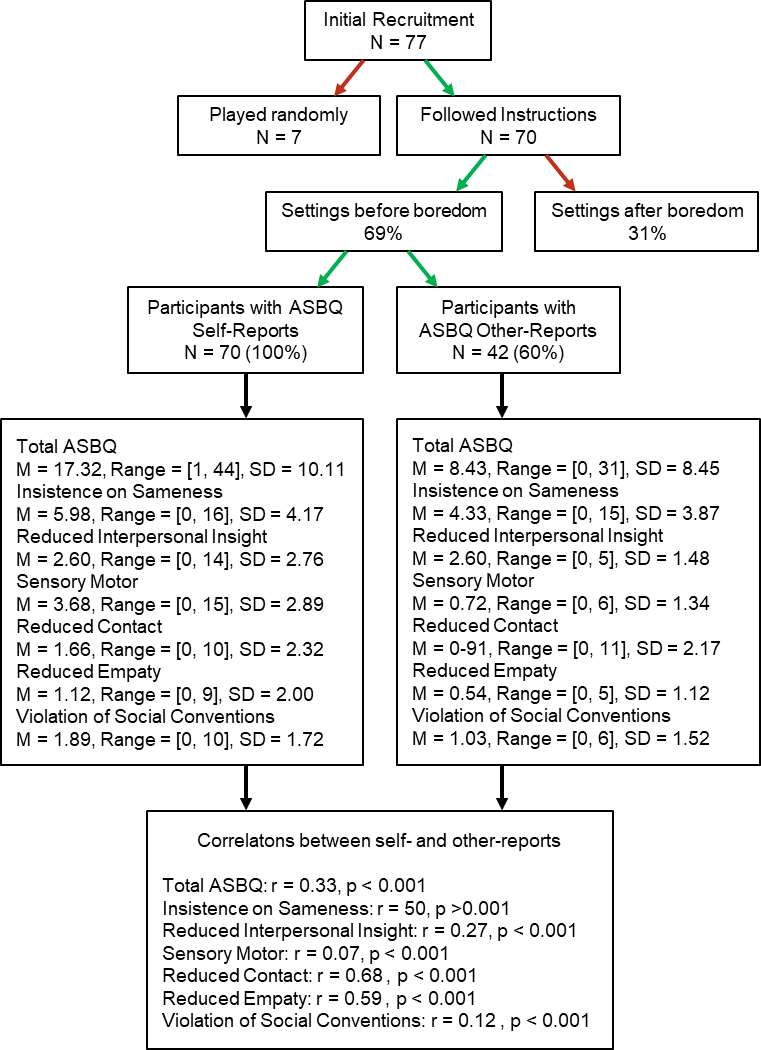
**

**Figure A. The preprocessing pipeline.** From the initial number of participants who were tested, seven were excluded because they did not follow the instructions. Of the remaining 70 participants, data from 31% percent of the settings were discarded because of boredom reports. All participants filled in the ASBQ self-reports, and 42 participants also had the other-reports filled in. The descriptive statistics and correlations between reports follow.

**Figure B. Participants’ predictions.** The mean (red dots) and standard deviation (red lines) of the participants’ predictions across every pattern of every environment are reported. The actual location of the animals is depicted in green. The model predictions based on the fit to the participants’ data is depicted in blue (pattern 1 = high drift, pattern 2 = high change-point probability, pattern 3 = high noise, pattern 4 = stable).


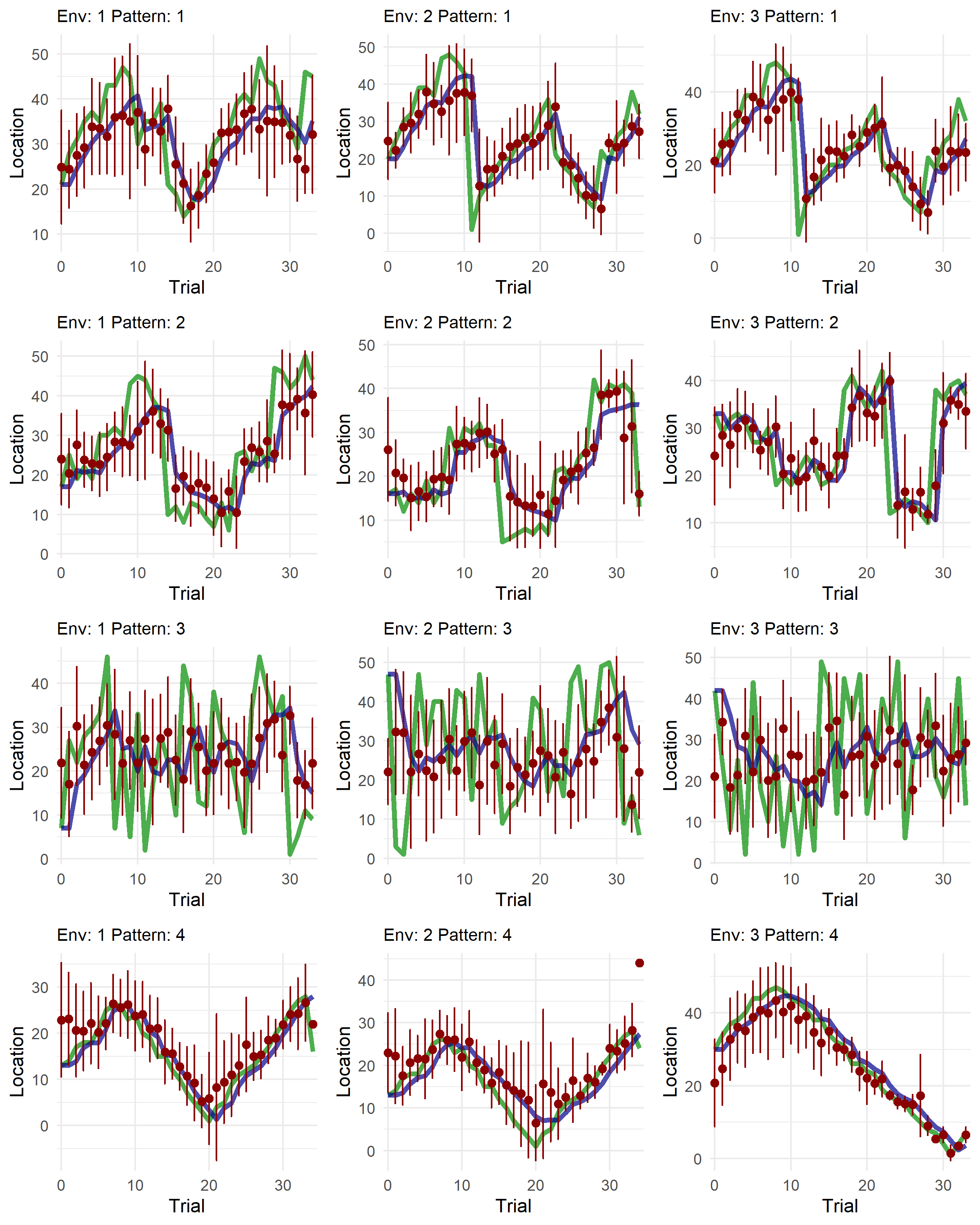


**
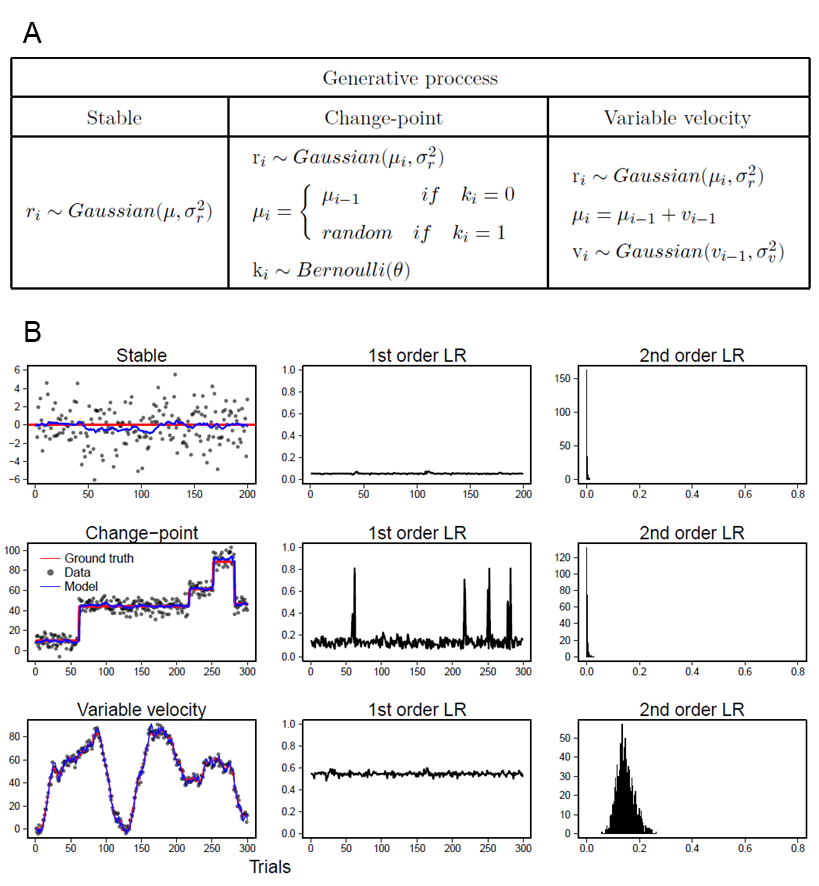
Figure C. Model simulations.** In order to test whether the model made accurate predictions, it was allowed to recover the generative processes detailed in (A). Markov chain Monte Carlo (MCMC) sampling implemented by JAGS [62] was used to approximate the posterior distributions of the parameters in the model. (B) shows the results of the simulation. In the left column the generative process and the mean of the model predictions are shown for each condition. The middle column corresponds to the posterior mean of the first-order learning rate on every trial. Finally, the right column shows the posterior samples of the second-order learning rate. It can be observed that the model accurately tracks the generative mean in the three environments. Additionally, it is evident that learning rates are sensitive to the type of environment the model is facing. In the condition without changes, the first-order learning rate stabilizes at low values allowing the model to filter out high levels of noise and to stay close to the mean. The second-order learning rate remains around zero in this condition, indicating that there was no rate of change affecting the outcomes. On the other hand, when the signal changes abruptly, the first-order learning rate remains stable and suddenly switches to high values when a change-point is found, indicating the need for quick adaptation. For this condition, the second-order learning rate still remains close to zero, accurately indicating the absence of a rate of change. When the signals changes at a variable velocity, the first-order learning rate stabilizes around a value related to the level of noise in the observations (like in the condition with no changes), but, interestingly, there is an activation of the second-order learning rate, indicating the presence of a rate of change in the outcomes, and the need for updating its values.


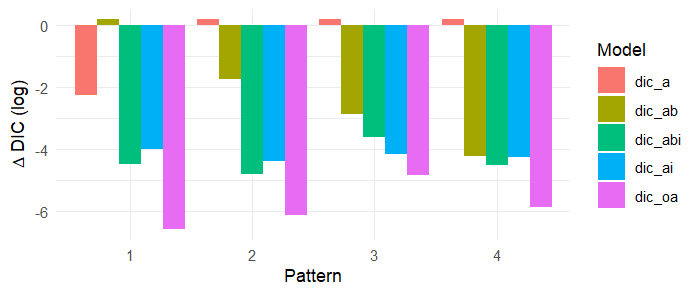

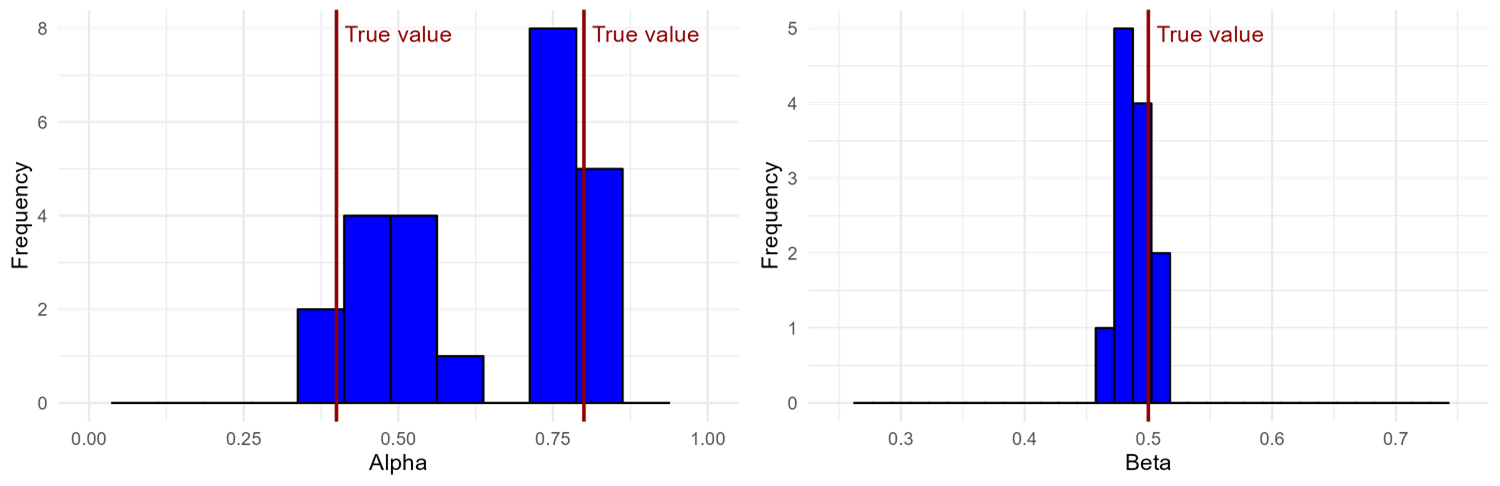
**Figure D. Parameters recovery.** We stimulated performance on all the task patterns (high change-point probability, high noise, stable, and high drift) of all settings (beach, sea, and grassland) for 30 artificial participants, fixing the values of the alpha parameters to 0.4 and 0.8, and the value of beta to 0.5. Then, we recovered the group-level parameters values from the simulations using the same fitting procedure that we used for the main analyses (in JAGS, using three chains with 20,000 samples each, where the first half of samples were discarded as burn-in). Both for alpha and for beta, the recovered values approximated the true values of the parameters, indicating successful parameters recovery.

**Figure E. Model comparison.** We compared the main model, which contains alpha and beta parameters fitted at the group level, to four additional models. Goodness of fit was measured using the Deviance Information Criterion (DIC) and the differences (delta) between the lowest-scoring (i.e., best) model and the other models are reported, with values closer to zero indicating better performance. We tested two reduced models, one with the two alpha parameters (but not beta) and one that contains only one alpha parameter. Given that the parameters were fitted at the group level, we also fitted these models in a hierarchical fashion, such that both group-level and individual-level parameters were estimated. Results of model comparison are reported separately for each pattern. The DIC for the model with alpha and beta parameters (dic_ab) was the best for the high-drift pattern, while the model with two alpha parameters (dic_a) was the best for the other three patterns (stable, high-noise, and high-change-point-probability). Given that the main model includes the alphas-only model (i.e., their fit is the same when beta = 0), these results indicate that the main model can capture performance on all patterns, but the alphas-only model fails when the drift (i.e., velocity) is high. For this reason, we decided to use the main model which includes both alphas and beta parameters. (dic_abi = model with alphas, beta, and fitted at the individual level, dic_ai = model with alphas, no beta, and fitted at the individual level, dic_oa = model with only one alpha, and no beta parameter).


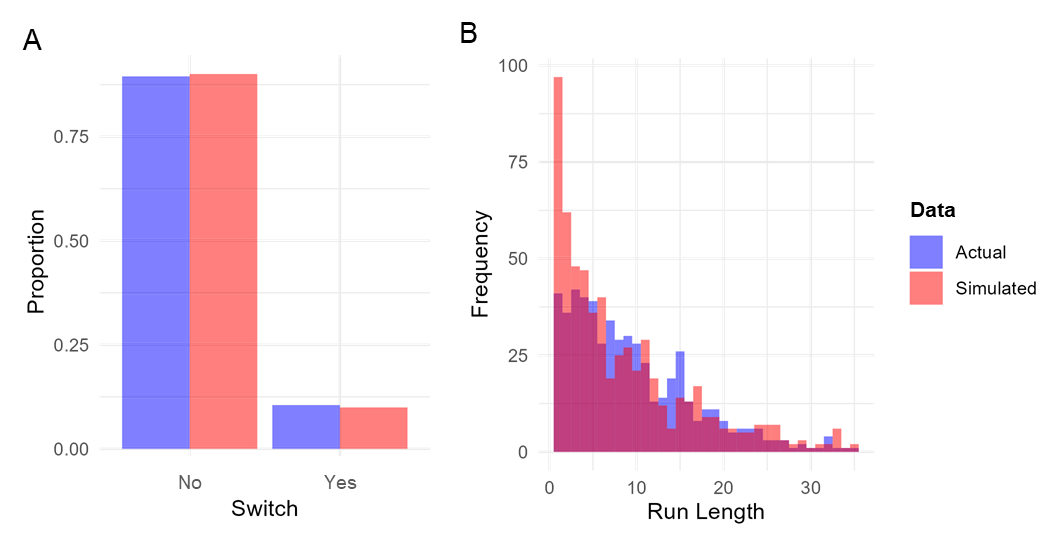
**Figure F. Comparing behavioral data to the predictions of the logistic model.** After fitting the logistic models with learning progress, expected prediction error, novelty and the other reports of insistence on sameness as covariates to the switch behavior (i.e., leave-stay decisions) of the participants, we used the model coefficients to simulate new data. The simulated data reflects well the original data, both in terms of proportion of leave vs stay decisions (A) and in terms of duration of run length (i.e., consecutive trials before switching) (B).

**Table A. Relation between leave-stay decisions and other-reports.*** indicates statistical significance

|  | Estimate | Std. Error | z-value | p-value |
| --- | --- | --- | --- | --- |
| Sensory stimulation and motor stereotypies | | | | |
| LP*Time*Trait | -0.063461 | 0.061018 | -1.040 | 0.29832 |
| PE*Time*Trait | -0.080255 | 0.068863 | -1.165 | 0.24385 |
| Reduced Interpersonal Insight | | | | |
| LP*Time*Trait | -0.084197 | 0.062975 | -1.337 | 0.18123 |
| PE*Time*Trait | -0.020433 | 0.061961 | -0.330 | 0.74157 |
| Reduced Contact | | | | |
| LP*Time*Trait | -0.19725 | 0.07881 | -2.503 | 0.0123 * |
| PE*Time*Trait | -0.13782 | 0.06694 | -2.059 | 0.0395 * |
| Reduced Empathy | | | | |
| LP*Time*Trait | -0.145428 | 0.061048 | -2.382 | 0.01721 * |
| PE*Time*Trait | -0.083442 | 0.052116 | -1.601 | 0.10936 |
| Violation of Social Conventions | | | | |
| LP*Time*Trait | -0.10725 | 0.05975 | -1.795 | 0.07264 |
| PE*Time*Trait | -0.04284 | 0.05097 | -0.841 | 0.40061 |

**Table B. Relation between leave-stay decisions and self-reports.**

* indicates statistical significance

|  | Estimate | Std. Error | z-value | p-value |
| --- | --- | --- | --- | --- |
| Sensory stimulation and motor stereotypies | | | | |
| LP*Time*Trait | 0.04130 | 0.04102 | 1.007 | 0.31402 |
| PE*Time*Trait | 0.06793 | 0.04203 | 1.616 | 0.10603 |
| Reduced Interpersonal Insight | | | | |
| LP*Time*Trait | -0.060214 | 0.036535 | -1.648 | 0.09933 |
| PE*Time*Trait | 0.011819 | 0.037038 | 0.319 | 0.74965 |
| Reduced Contact | | | | |
| LP*Time*Trait | -0.02196 | 0.04330 | -0.507 | 0.612083 |
| PE*Time*Trait | -0.08396 | 0.03910 | -2.147 | 0.031761 * |
| Reduced Empathy | | | | |
| LP*Time*Trait | -0.073846 | 0.041345 | -1.786 | 0.07408 |
| PE*Time*Trait | -0.016536 | 0.035128 | -0.471 | 0.63783 |
| Violation of Social Conventions | | | | |
| LP*Time*Trait | -0.010826 | 0.037005 | -0.293 | 0.76986 |
| PE*Time*Trait | -0.005329 | 0.032709 | -0.163 | 0.87058 |

**Appendix B. Supplementary Analyses.**

**Effect of pattern type on leave-stay decisions.**

A logistic model was run with leave-stay decisions as dependent variable, and pattern type and trial number as predictors. Random intercepts for participant, environment, and animal type were added. As expected, we find a significant effect of trial number, with greater trial number relating to increased probability of leave decision (F = 123.52) and no effect of pattern type (F = 2.05).

**Relation between exploratory decisions and self- and other-reports.**

Logistic models were run both on self-reports and other-reports, for each scale of the ASBQ questionnaire. Participants were divided in two groups, depending on the mean value of each scale (i.e., above the mean and below the mean). Here, significant results are reported. A full description of analyses can be found on OSF:

https://osf.io/h2prm

Self-reports of reduced contact showed a significant interaction with novelty (low group: β = .60, SE = .13, *p* < 0.001, high group: β = .57, SE = .18, *p* = 0.001) and expected prediction error (low group: β = -.17, SE = .09, *p* = 0.048, high group: β = -.09, SE = .13, *p* = .52) in predicting exploratory choices; similar results were found for novelty (low group: β = .69, SE = .15, *p* < 0.001, high group: β = .57, SE = .15, *p* < 0.001) and expected learning progress (low group: β = -.18, SE = .10, *p* = 0.07, high group: β = .21, SE = .10, *p* = 0.044) in interaction with self-reports of reduced social interactions. Regarding other-reports, we saw similar results for novelty (low group: β = .82, SE = .19, *p* < 0.001, high group: β = .59, SE = .22, *p* = 0.008) and expected learning progress (low group: β = -.14, SE = .12, *p* = .25, high group: β = .38, SE = .18, *p* = .03) for the full scale of autistic traits.
