# Supplementary material for: Autistic traits foster effective curiosity-driven exploration
Source: PLoS Comput Biol. 2024 Oct 31;20(10):e1012453. doi: 10.1371/journal.pcbi.1012453 (PMC11527316; doi:10.1371/journal.pcbi.1012453)
Supplement: Attachment — Submitted filename: Review_PCOMPBIOL-D-23-01580.pdf [file pcbi.1012453.s002.pdf]

Title: Autistic traits foster effective curiosity-driven exploration

Review comments:

This study by Poli et al. investigates individual variability in curiosity-driven explorative behavior, in its relation to autistic traits using self- and other-report measures. To assess the learning strategies of the participants, the study extends the hierarchical reinforcement learning (RL) model previously developed by Poli et al. (2022) to quantify key constructs such as prediction error, learning progress, and expected values of these. Using a dataset collected from 77 participants, the study employed multiple logistic regression models to predict leave-stay decisions (or explored options) as a function of autistic traits and (expected) prediction error/learning progress. The major finding of this study is that participants with higher-than average scores of a particular autistic trait (i.e., insistence-on-sameness) were less likely to explore early in the task and eventually relied on learning progress toward the end of the task, resulting in a better performance.

Altogether, the study addresses an interesting and important question related to individual variability, autistic traits, and their influence on intrinsic drive to learn. The key novelty is the investigation of autistic traits under the unique task design which allows self-paced learning in the absence of explicit rewards. That being said, the manuscript would benefit from some clarifications and control analysis, especially regarding the hierarchical RL model and major analyses. Below are some points for suggestions for improving the manuscript.

Major comments:

1. Evidence for utility of the hierarchical Bayesian model

All of the major analyses in this study heavily relies on the hierarchical Bayesian model, which yields key estimates for each participant – such as (expected) prediction error and learning progresses – that are later used for regression analyses. Therefore, I feel that the authors need to more explicitly establish the utility or superiority of this model in comparison to at least one other competing model, as an alternative hypothesis for how the subjects might learn about the task environment. To put it in another way, the major focus of this study is individual variability in learning strategies, yet the hierarchical RL model assumes that the given latent structures and their updates are commonly undergoing for all participants. Could the authors provide further evidence that could support this?

Specifically, since the participants are not explicitly aware that some animals are “drifting” in their true position, I wonder if adding the update mechanism for tracking the drift in the animals’ movement (V’) truly helps with the fit for all participants, or whether there might be a subset of participants that do not employ this mechanism. If you could obtain goodness-of-fit for each participant for each model, this would be a simple comparison of how many subjects are better explained by the full model.

Moreover, related to the point about individual variability, I was a little surprised to learn that learning rates were fitted across participants (to improve the stability of fit). I am concerned if this goes against the purpose of capturing behavioral aspects of individual variability with the model. Is this because fitting the data for each participant reduces the quality of fit? I assume other parameters (than learning rates) were fitted to each subject individually, but this could be clarified.

2. Some clarification figures and analysis

The study refers to increased performance for high insistence-on-sameness groups. But in general, it's hard to grasp intuition on the task structure and how each participant performed the task, and what the performance means for each subject. Some clarification questions first, as I'm a little confused about the task structure: if one animal is chosen to reveal their locations, are the other animals also moving according to their own dynamics without being seen, or do they remain unchanged from their previously revealed locations? Regarding this, I think it would greatly help the readers to provide plots for an example session for example participants (each in -1SD and +1SD group), perhaps as additional panels in Figure 2 or as a separate figure. (For example, it could plot the position of each chosen animal in its respective colors, overlaid with participant's responses.)

Also, the current conclusion about increased performance for higher insistence-on-sameness score mainly comes from more nuanced group-level analysis (i.e., comparing -1SD and +1SD groups in Section 3.3.). I wonder if this could be made a more general claim by looking at the trend in all of the participants. Could authors provide a supplementary analysis for a simple correlation (or regression) between insistence-on-sameness and overall performance in each subject?

Lastly, related to Table 1 and Figure 3, was there any significant difference between high vs. low group (+1SD vs. -1SD in insistence-on-sameness score) in terms of the goodness-of-fit in the logistic regression model? Hopefully we would not want any significant difference between them for fair comparison, but if there is one it would be important to report it.

3. Reaction time analysis

As this study concerns motivation and intrinsic drive to learn, I think the manuscript would also benefit greatly by studying reaction time (RT), which is a good indicator of motivation. Would it be possible to perform regression analyses on RT, similar to logistic regression on P(Leave) that the authors have performed? If the authors could confirm similar effects on RT, this would be a good supporting evidence to the claims made in the paper.

Minor comments:

1. Figure 1 A-E: the instructions in the figures are too small to be read. I understand that these are the true scale of the task that the participants performed, but could you increase the font so that readers can also see the instructions in the figure?

2. The current setting for hierarchical RL assumes that there is one model corresponding to each of the four animals in the task environment. Here, is it assuming that only the model for the chosen animal is updated, while the rest remains fixed?

3. In Section 2.4, what was the proportion of trials that were discarded for reported boredom? I think this quantity is important to report here. Also it might be worth checking if this percentage (for each subject) is correlated with the insistence-on-sameness score, which is the key measure in this study.

4. In Section 2.5, was the motivation for introducing the z_t variable (with Bernoulli distribution) to allow switching between learning about abrupt vs. gradual changes (with high & low learning rates respectively)? More clarification (in the paragraph after equations) could help here. Also, I assume B is the response but this is also not specified in the paragraph.

5. The definition of novelty (N_t) and its fixed parameter (k) could also help from more clarifications. Since this was not part of the RL model fitted to participants' behavior, how was k set in the analysis?

6. I assume "stay-leave decisions" in Section 3-1 means whether the subjects chose the same animal from the previous trial. This could be defined explicitly.

7. There is a mismatch between Equation for B_{i+1} and B_{at+1} in Figure 3: noise is η in the former and $1/\eta$ in the latter.

8. Figure 4 (and its caption) could be made a little more intuitive. Insistence on Sameness is below the bottom panel, but this could be moved to top since it appears on all panels. Also, I think the distinction between red vs. blue line is high vs. low score, so this could be labeled as such in the legend (e.g. high (+1SD) and low (-1SD)).

Other minor edits/typos:

1. Please consider adding equation numbers for all the equations, as it's much easier to refer to them and is considered a standard practice for computational works.
2. In Section 3-1, right after the equation for P(Leave$_t$): Where → where
3. Figure 3 caption: I think double-boarded nodes are stochastic?
4. Table 1 caption: All terms <u>where</u> in interaction … → All terms <u>were</u> in interaction?
