# Supplementary material for: Autistic traits foster effective curiosity-driven exploration
Source: PLoS Comput Biol. 2024 Oct 31;20(10):e1012453. doi: 10.1371/journal.pcbi.1012453 (PMC11527316; doi:10.1371/journal.pcbi.1012453)
Supplement: Attachment — Submitted filename: Reviews_AutisticTraits_Poli_et_al_v2.docx [file pcbi.1012453.s003.docx]

Reviewer #1

Review comments:

This study by Poli et al. investigates individual variability in curiosity-driven explorative behavior, in its relation to autistic traits using self- and other-report measures. To assess the learning strategies of the participants, the study extends the hierarchical reinforcement learning (RL) model previously developed by Poli et al. (2022) to quantify key constructs such as prediction error, learning progress, and expected values of these. Using a dataset collected from 77 participants, the study employed multiple logistic regression models to predict leave-stay decisions (or explored options) as a function of autistic traits and (expected) prediction error/learning progress. The major finding of this study is that participants with higher-than average scores of a particular autistic trait (i.e., insistence-on-sameness) were less likely to explore early in the task and eventually relied on learning progress toward the end of the task, resulting in a better performance.

Altogether, the study addresses an interesting and important question related to individual variability, autistic traits, and their influence on intrinsic drive to learn. The key novelty is the investigation of autistic traits under the unique task design which allows self-paced learning in the absence of explicit rewards. That being said, the manuscript would benefit from some clarifications and control analysis, especially regarding the hierarchical RL model and major analyses. Below are some points for suggestions for improving the manuscript.

Major comments:

1. Evidence for utility of the hierarchical Bayesian model

All of the major analyses in this study heavily relies on the hierarchical Bayesian model, which yields key estimates for each participant – such as (expected) prediction error and learning progresses – that are later used for regression analyses. Therefore, I feel that the authors need to more explicitly establish the utility or superiority of this model in comparison to at least one other competing model, as an alternative hypothesis for how the subjects might learn about the task environment. To put it in another way, the major focus of this study is individual variability in learning strategies, yet the hierarchical RL model assumes that the given latent structures and their updates are commonly undergoing for all participants. Could the authors provide further evidence that could support this?

Specifically, since the participants are not explicitly aware that some animals are “drifting” in their true position, I wonder if adding the update mechanism for tracking the drift in the animals’ movement (V’) truly helps with the fit for all participants, or whether there might be a subset of participants that do not employ this mechanism. If you could obtain goodness-of-fit for each participant for each model, this would be a simple comparison of how many subjects are better explained by the full model. Moreover, related to the point about individual variability, I was a little surprised to learn that learning rates were fitted across participants (to improve the stability of fit). I am concerned if this goes against the purpose of capturing behavioral aspects of individual variability with the model. Is this because fitting the data for each participant reduces the quality of fit? I assume other parameters (than learning rates) were fitted to each subject individually, but this could be clarified.

We thank the reviewer for this insightful comment. We agree that model comparison is needed, and we have now set up multiple competing models and compared their performance using the Deviance Information Criterion (DIC). The original model allowed for 3 different types of learning: 1. Slow updating; 2. Fast updating; 3. Velocity estimation. The first two types of learning were regulated by two alpha parameters, one taking lower values (slow updating) and one taking greater values (fast updating), while velocity estimation was regulated by the beta parameter. We now have two reduced models, one with the two alphas (but not beta) and one that contains only one alpha. In addition, given that the parameters were fitted at the group level, we also fitted these models in a hierarchical fashion, such that both group-level and individual-level parameters were estimated. The results of model comparison are now specified in the supplementary materials:

***Figure S5. Model comparison.*** *We compared the main model, which contains alpha and beta parameters fitted at the group level, to four additional models. Goodness of fit was measured using the Deviance Information Criterion (DIC) and the differences (delta) between the lowest-scoring (i.e., best) model and the other models are reported, with values closer to zero indicating better performance. We tested two reduced models, one with the two alpha parameters (but not beta) and one that contains only one alpha parameter. Given that the parameters were fitted at the group level, we also fitted these models in a hierarchical fashion, such that both group-level and individual-level parameters were estimated. Results of model comparison are reported separately for each pattern. The DIC for the model with alpha and beta parameters (dic_ab) was the best for the high-drift pattern, while the model with two alpha parameters (dic_a) was the best for the other three patterns (stable, high-noise, and high-change-point-probability). Given that the main model includes the alphas-only model (i.e., their fit is the same when beta = 0), these results indicate that the main model can capture performance on all patterns, but the alphas-only model fails when the drift (i.e., velocity) is high. For this reason, we decided to use the main model which includes both alphas and beta parameters. (dic_abi = model with alphas, beta, and fitted at the individual level, dic_ai = model with alphas, no beta, and fitted at the individual level, dic_oa = model with only one alpha, and no beta parameter).*

It must be noted that on instances in which the model containing only alphas is best, the full model containing both alphas and beta performs equally well, with values of beta that approximate zero. In other words, the full model collapses to the reduced model in some situations, but not when beta is needed.

2. Some clarification figures and analysis

The study refers to increased performance for high insistence-on-sameness groups. But in general, it’s hard to grasp intuition on the task structure and how each participant performed the task, and what the performance means for each subject. Some clarification questions first, as I’m a little confused about the task structure: if one animal is chosen to reveal their locations, are the other animals also moving according to their own dynamics without being seen, or do they remain unchanged from their previously revealed locations? Regarding this, I think it would greatly help the readers to provide plots for an example session for example participants (each in -1SD and +1SD group), perhaps as additional panels in Figure 2 or as a separate figure. (For example, it could plot the position of each chosen animal in its respective colors, overlaid with participant’s responses.)

We now state explicitly this aspect of the animals’ dynamics in the methods section:
*“These patterns were independent from each other, such that while the participant is playing with one of the animals, the locations of the other animals remain unchanged.”*

We thank the reviewer for the suggestion to include a plot with an example session. We fully agree that the task has many aspects, and the modelling also complicates things. Showing everything together is the best way to convey the connection between the participants’ predictions, the model fitted to them, and the moments in time when the participant has switched animal (i.e., the dependent variable). We would like to point out that model comparison preferred a model with parameters at the group level, but not at the individual level. Hence, we have no evidence that alpha and beta parameters differed as a function of autistic traits. What differed across traits scores were the factors that led participants to leave an environment and pick a different one, which is harder to represent in this plot. Hence, we added a plot with an example session to Figure 2, but we keep the comparisons across autistic traits for the results section:

***Figure 2. A.*** *The hiding patterns were generated from Gaussian distributions, varying their drift, their change-point probability (CPP), and their standard deviation (i.e., noise). This results in four different patterns, three of which are learnable (i.e., all but the high noise pattern).* ***B.*** *Example performance from one participant.* *Participants guessed the location of each animal (coloured dots) and received feedback about its actual location (grey line). The delta-rule model was inferring the beliefs of the participants (coloured lines) from their predictions (the dots). Learning progress, prediction errors, and novelty were computed for all patterns in the same way.*

Also, the current conclusion about increased performance for higher insistence-on-sameness score mainly comes from more nuanced group-level analysis (i.e., comparing -1SD and +1SD groups in Section 3.3.). I wonder if this could be made a more general claim by looking at the trend in all of the participants. Could authors provide a supplementary analysis for a simple correlation (or regression) between insistence-on-sameness and overall performance in each subject?

We apologize for the misunderstanding about these analyses. Autistic traits were included as a continuous variable, but dichotomized in the figure to aid interpretability. This is now clearly stated both in the text and in the caption of Figure 6:

*Autistic traits were treated as a continuous variable, but they are dichotomized in the figure to aid interpretability.*

Lastly, related to Table 1 and Figure 3, was there any significant difference between high vs. Low group (+1SD vs. -1SD in insistence-on-sameness score) in terms of the goodness-of-fit in the logistic regression model? Hopefully we would not want any significant difference between them for fair comparison, but if there is one it would be important to report it.

As in the previous comment, autistic traits were treated as a continuous variable. This is now stated explicitly both in the text and in the caption of Figure 4.

3. Reaction time analysis

As this study concerns motivation and intrinsic drive to learn, I think the manuscript would also benefit greatly by studying reaction time (RT), which is a good indicator of motivation. Would it be possible to perform regression analyses on RT, similar to logistic regression on P(Leave) that the authors have performed? If the authors could confirm similar effects on RT, this would be a good supporting evidence to the claims made in the paper.

We have taken up the reviewer’s suggestion and run the following exploratory analysis for the RTs:

```
log(RT) ~ LP + EPE + novelty)*Time*InsSameASBQ_oth +
	    (1|subj) + (1|env) + (1|char)+ (1|pattern)

FIXED EFFECTS:
---------------------------------------------------------------------------
                                      Est.   S.E.   t val.      d.f.      p
---------------------------------- ------- ------ -------- --------- ------
(Intercept)                           0.16   0.02     7.03     37.06   0.0
LP                                    0.00   0.00     0.54   5588.30   0.5
EPE                                   0.00   0.00     0.83     80.51   0.4
novelty                               0.02   0.00     9.09   6233.14   0.0
Time                                  0.00   0.00     1.73   5179.53   0.0
InsSameASBQ_oth                       0.00   0.02     0.12     39.17   0.9
LP:Time                               0.00   0.00     0.67   6305.96   0.5
EPE:Time                              0.01   0.00     4.79   6156.75   0.0
novelty:Time                         -0.00   0.00    -1.61   6210.37   0.1
LP:InsSameASBQ_oth                   -0.00   0.00    -1.35   6303.39   0.1
EPE:InsSameASBQ_oth                   0.00   0.00     1.61   6033.15   0.1
novelty:InsSameASBQ_oth               0.00   0.00     1.12   6314.60   0.2
Time:InsSameASBQ_oth                  0.00   0.00     0.98   6326.55   0.3
LP:Time:InsSameASBQ_oth              -0.00   0.00    -0.04   6302.93   0.9
EPE:Time:InsSameASBQ_oth              0.00   0.00     0.06   6232.25   0.9
novelty:Time:InsSameASBQ_oth          0.00   0.00     1.15   6311.99   0.2
--------------------------------------------------------------------------
```

The effects of interest (LP:Time:InsSameASBQ_oth and EPE:Time:InsSameASBQ_oth) were not significant. We believe that RTs are indeed a very interesting measure, but it might capture variability due to many different factors (e.g., someone might go fast because the task is easy, or because they are engaged, or because they want to finish more quickly and they do not care about accuracy as much). Hence, we prefer not to include analyses of RTs in the manuscript.

Minor comments:

1. Figure 1 A-E: the instructions in the figures are too small to be read. I understand that these are the true scale of the task that the participants performed, but could you increase the font so that readers can also see the instructions in the figure?

Instructions have been added below each figure in bigger characters.

2. The current setting for hierarchical RL assumes that there is one model corresponding to each of the four animals in the task environment. Here, is it assuming that only the model for the chosen animal is updated, while the rest remains fixed?

Yes, that is correct. We have now stated this in the manuscript (p. 11):

*“A model was fit for each animal of each setting independently. Hence, at every trial, only the model of the animal that is selected is updated, while the others remain the same.”*

3. In Section 2.4, what was the proportion of trials that were discarded for reported boredom? I think this quantity is important to report here. Also it might be worth checking if this percentage (for each subject) is correlated with the insistence-on-sameness score, which is the key measure in this study.

We have now reported how many settings were included in the analysis in the methods section (and supplementary figure S1):

*From the included participants, we discarded the data from the settings following the moment they reported boredom. Boredom occurred after the 1^st^ setting for 21 participants, after the 2^nd^ setting for 23 participants, and after the 3^rd^ setting for 26 participants. These data were discarded before running any analysis, including the hierarchical delta-rule model. This decision was motivated by the fact that the main focus of this research was intrinsically motivated exploration, which cannot be measured in conditions of boredom.*

We checked whether the setting number in which participants reported boredom was related to their insistence on sameness. There was no significant effect both for self-reports (p = 0.36) and other-reports (p = 0.90).

4. In Section 2.5, was the motivation for introducing the zt variable (with Bernoulli distribution) to allow switching between learning about abrupt vs. gradual changes (with high & low learning rates respectively)? More clarification (in the paragraph after equations) could help here. Also, I assume B is the response but this is also not specified in the paragraph.

We thank the reviewer for pointing out that some aspects of the model were unclear. The explanation of these parameters has been re-written, both in the text (Section 2.5) and in the caption of Figure 3.

5. The definition of novelty (Nt) and its fixed parameter (k) could also help from more clarifications. Since this was not part of the RL model fitted to participants’ behavior, how was k set in the analysis?

Originally, k was a free parameter. However, in the manuscript we always considered it as a fixed parameter, where k = 1, which is equivalent to not having k at all. For this reason, we now removed any mention of k:

*“The novelty of each option at any given trial was computed as an inverse function of exposure to that option, hence:*

$$N_{t}=-t$$

*Where* $t$ *is the number of trials played with the same animal.”*

6. I assume “stay-leave decisions” in Section 3-1 means whether the subjects chose the same animal from the previous trial. This could be defined explicitly.

We thank the reviewer for pointing out that this dependent variable was never clearly defined. We now define all variables extracted from the participants’ behavior during the task in the methods section:

*We extracted three key variables from this task. First, participants’ predictions about where the character would appear were extracted for each trial. This variable is the observable state that is used to fit the delta-rule model (see Figure 2B and the Methods section). Second, for each trial we measured whether participants chose a new animal or stayed with the same one. These are called leave-stay decisions. Finally, every time a leave decision is made and a new animal is chosen, we measured which animal was chosen among the three available options. These are called exploratory decisions. Leave-stay decisions and exploratory decisions are the main dependent variables of our paper, as we seek to understand when participants decide to explore, and what they decide to explore.*

7. There is a mismatch between Equation for Bi+1 and Bat+1 in Figure 3: noise is 𝜼 in the former and 1/𝜼 in the latter.

The mismatch in equation (3) has now been corrected.

8. Figure 4 (and its caption) could be made a little more intuitive. Insistence on Sameness is below the bottom panel, but this could be moved to top since it appears on all panels.

Also, I think the distinction between red vs. blue line is high vs. low score, so this could be labeled as such in the legend (e.g. high (+1SD) and low (-1SD)).

Figure 4 has been updated accordingly.

Other minor edits/typos:

1. Please consider adding equation numbers for all the equations, as it’s much easier to refer to them and is considered a standard practice for computational works.

2. In Section 3-1, right after the equation for P(Leavet): Where → where

3. Figure 3 caption: I think double-boarded nodes are stochastic?

4. Table 1 caption: All terms where in interaction … → All terms were in interaction?

Equations numbers and typos have been adjusted accordingly. For Figure 3, we have used the notation described in Lee (2018), where double-boarded nodes represent deterministic variables. Here, V and V’ are deterministic because their value can be computed exactly given the knowledge of the other parameters values (alpha, beta, and z, and phi).

Reviewer #2

The authors report on whether individual differences in behavior in a patch foraging task can be related to autistic self-report measures. The task is cleverly designed, and the modeling of the behavior is quite extensive. However, the overall value of the computational modeling of behavior is lessened by many unexplained and confounding choices in the data analytic strategy and the authors' inability to ground their modeling in basic behaviors. Overall, the study seems underpowered to reliably assess how individual differences in autistic traits correlate with curiosity-driven exploration.

1. Please provide more details in terms of the percentage of trials thrown away for the included participants based on the boredom exclusion criteria. Also, the authors use logistic models to predict when participants abandoned choosing a particular animal and switched to another animal; these rely on trial-by-trial estimates. Were the trials overlapping with boredom not included in fitting the hierarchical reinforcement learning model, or were they simply censored? Overall, there appears to be a great deal of data curation, which is concerning in relation to the relatively small effect sizes reported when the task behavior is related to the autism-related self-reports.

We now provide this information in the methods section. We also state explicitly that the data was excluded before fitting both the reinforcement-learning model and the logistic models:

*Participants who indicated that they did not understand the task instructions were excluded from the analyses (N=7).* *From the included participants, we discarded the data from the settings following the moment they reported boredom. Boredom occurred after the 1^st^ setting for 21 participants, after the 2^nd^ setting for 23 participants, and after the 3^rd^ setting for 26 participants. These data were discarded before running any analysis, including the hierarchical delta-rule model. This decision was motivated by the fact that the main focus of this research was intrinsically motivated exploration, which cannot be measured in conditions of boredom.*

In addition, we now explain all our decisions step by step in a new supplementary Figure S1:

***Figure S1. The preprocessing pipeline.*** *From the initial number of participants who were tested, seven were excluded because they did not follow the instructions. Of the remaining 70 participants, data from 31% percent of the settings were discarded because of boredom reports. All participants filled in the ASBQ self-reports, and 42 participants also had the other-reports filled in. The descriptive statistics and correlations between variables are reported for these separate analysis steps.*

Data curation is indeed greater than what can be seen in learning tasks with similar features (Nassar & Troiani, 2021). This is due to the unique nature of this task, that is, the free exploration it allows. We now address this important point in the discussion:

*When studying the active aspects of learning, researchers need to find ways of handing control over to the participants without losing experimental rigor. This makes this type of research different from other areas of cognitive science, where paradigms are mostly passive, and participants are required to follow instructions (e.g., O’Reilly et al., 2013). Although this approach comes with unique benefits (i.e., studying aspects of behavior that are driven by intrinsic motivation and engagement), it also involves greater data curation. Future studies should include bigger samples to assess how intrinsically motivated behaviors change as a function of these preprocessing decisions.*

2. Another important caveat is that the authors only found a relationship between the model-derived reinforcement learning (RL) estimates and the other-reports. Substantially weaker or null effects were found when the same RL measures were correlated with self-reports. This is concerning because the authors report that only 60% (42 out of the 70 participants) handed in the other self-report questionnaire, and therefore the sample sizes are very different.

We acknowledge that this is a limitation of our study. We tried to be as transparent as we could, showing the results for self- and other-reports side by side. It must be noted that for the results that are not significant, the direction of the effect is always the same across reports. Although in the manuscript we refrain from interpreting null results, this pattern of effects are somewhat consistent across self- and other-reports. Moreover, within the 42 participants who filled in the other-report questionnaire, we preserve the same variability in autistic traits, although with a smaller sample size. The trade-off between self- and other-reports is a difficult one, because self-reports are easier to acquire, but also likely to be less reliable. Conversely, other-reports are more difficult to acquire: we cannot compel participants to hand them in, as they depended on the cooperation of their parents. On the other side, other-reports may work especially well for participants that score high in autistic traits because individuals with ASD may have difficulty reflecting and reporting on their own behavioral and emotional characteristics or may mask them. We now justify our decision in the methods section:

*“In the analyses, we primarily focused on other-reports collected from a parental figure. This decision was made based on the fact that individuals who have stronger autistic traits may also have more difficulty reflecting and reporting on their behavioral and emotional patterns or they may mask their patterns, which may result in more unreliable self-reports (Pearl et al., 2016). Indeed, previous research has found discrepancies between self- and other-reports (Lerner et al., 2012). For this reason, we expected parental reports to be more reliable. However, we always present results both for parental-reports and self-reports.”*

3. The novelty of each option utilizes kappa as a fixed parameter, but the value of this parameter and how it was determined is not explained. This is important since the best-fitting model includes trial-by-trial novelty estimates.

Originally, kappa was a free parameter. However, in the manuscript we always considered it as a fixed parameter, where k = 1, which is equivalent to not having kappa at all. For this reason, we now removed any mention of kappa:

*“The novelty of each option at any given trial was computed as an inverse function of exposure to that option, hence:*

$$N_{t}=-t$$

*Where* $t$ *is the number of trials played with the same animal.”*

4. There is no basic presentation of the participants' behaviors within the task and how they scale with individual difference measures. We must rely entirely on the authors' statement that the behavior was well fit without any direct evidence. I would like to see some basic behaviors plotted in relation to key decision criteria in the task, such as the frequency of switching between animals, the duration of sampling within a patch (i.e., animal), etc., and then how well the model can predict those behaviors (independent of the individual differences). Otherwise, it is very difficult to judge the validity of the modeling in relation to the individual differences.

We thank the reviewer for pointing this out, and we agree that behavioral data should be plotted directly, and also in comparison to the model. We also would like to point out that the fitting to switching behavior and duration of sampling was not made as part of the reinforcement-learning model, but only in a second instance through logistic regression models. The reinforcement-learning model was fitted to the predictions of the participants (i.e., their guesses about where the animals were hiding) to infer their beliefs about the animals’ locations. Hence, also the plotting of raw data in comparison to the reinforcement-learning and logistic models has to be differentiated accordingly. Regarding the reinforcement-learning model, we now plot the performance of one example participant and the model estimates of their beliefs in Figure 2B:

***Figure 2. A.*** *The hiding patterns were generated from Gaussian distributions, varying their drift, their change-point probability (CPP), and their standard deviation (i.e., noise). This results in four different patterns, three of which are learnable (i.e., all but the high noise pattern).* ***B.*** *Participants guessed the pattern of each animal (coloured dots) and received feedback about their actual location (grey line). The delta-rule model was inferring the beliefs of the participants (coloured lines) from their predictions (the dots). Learning progress, prediction errors, and novelty were computed for all patterns in the same way.*

Regarding the logistic regression, we now plot the actual data and the data predicted by the model in supplementary figure S6:

***Figure S6. Comparing behavioral data to the predictions of the logistic model.*** *After fitting the logistic models with learning progress, expected prediction error, novelty and the other reports of insistence on sameness as covariates to the switch behavior (i.e., leave-stay decisions) of the participants, we used the model coefficients to simulate new data. The simulated data reflects well the original data, both in terms of proportion of leave vs stay decisions (A) and in terms of duration of run length (i.e., consecutive trials before switching) (B).*

Reviewer #3: PLos Review

I thank the authors for submitting this paper, which was read with great interest. Poli et al., make use of a free choice exploration task in which participants choose to learn about the hidden location of a set of four animals across different contexts, whose location were determined by a distinct (learnable) path. The authors have done a good job of bringing attention to the understanding of autistic traits (insistence on sameness) on learning and free choice exploration and I agree with the use of modelling approaches to understand the computational signatures of these traits, and the emphasis on understanding individual differences in these traits as opposed to diagnosis. However, there are some issues that limit my enthusiasm for this manuscript, which should be addressed before the manuscript would be considered suitable for publication.

Major points

1. Computational modelling: Given that in its current form, results of the manuscript hinge on the output of the computational modelling, I want to draw attention to a number of issues that we had with the modelling itself:

a. My primary concern is that whilst the model has been used in previous work from the same group, we would ask the authors to include some model quality check given current guidelines to aid in the reliability, interpretation and identifiability of parameters (see Wilson & Collins 2019 eLife, Palminteri et al 2017 TICS). For example, starting with simulating some data (as undertaken in previous work) to see if the key behavioural features of the task can be recapitulated (see point d. below), but also demonstrating the recoverability of the parameters. It is also conceivable that despite the sophistication of the model used in the paper, a simpler model may be able to explain the data similarly well. As such, I would recommend the inclusion of model comparisons.

We thank the reviewer for raising these important points. Here is how we addressed them:

- Data simulation: We generated three patterns, and checked the model’s ability to recover key features of these patters (i.e., abrupt change points, drift, etc.). This is now represented in supplementary figure S3:

***Figure S3. Model simulations.*** *In order to test whether the model made accurate predictions, it was allowed to recover the generative processes detailed in (A). Markov chain Monte Carlo (MCMC) sampling implemented by JAGS (Plummer, 2003) was used to approximate the posterior distributions of the parameters in the model. (B) shows the results of the simulation. In the left column the generative process and the mean of the model predictions are shown for each condition. The middle column corresponds to the posterior mean of the first-order learning rate on every trial. Finally, the right column shows the posterior samples of the second-order learning rate. It can be observed that the model accurately tracks the generative mean in the three environments. Additionally, it is evident that learning rates are sensitive to the type of environment the model is facing. In the condition without changes, the first-order learning rate stabilizes at low values allowing the model to filter out high levels of noise and to stay close to the mean. The second-order learning rate remains around zero in this condition, indicating that there was no rate of change affecting the outcomes. On the other hand, when the signal changes abruptly, the first-order learning rate remains stable and suddenly switches to high values when a change-point is found, indicating the need for quick adaptation. For this condition, the second-order learning rate still remains close to zero, accurately indicating the absence of a rate of change. When the signals changes at a variable velocity, the first-order learning rate stabilizes around a value related to the level of noise in the observations (like in the condition with no changes), but, interestingly, there is an activation of the second-order learning rate, indicating the presence of a rate of change in the outcomes, and the need for updating its values.*

- Parameter recovery: We fixed the values of alpha and beta parameters, simulated data, and then fitted the model to these simulated data to check whether the original parameters values could be recovered. This is represented now in supplementary figure S4:

***Figure S4. Parameters recovery.*** *We stimulated performance on all the task patterns (high change-point probability, high noise, stable, and high drift) of all settings (beach, sea, and grassland) for 30 artificial participants, fixing the values of the alpha parameters to 0.4 and 0.8, and the value of beta to 0.5. Then, we recovered the group-level parameters values from the simulations using the same fitting procedure that we used for the main analyses (in JAGS, using three chains with 20,000 samples each, where the first half of samples were discarded as burn-in). Both for alpha and for beta, the recovered values approximated the true values of the parameters, indicating successful parameters recovery.*

- Model comparison: We have now added four additional variations of the original model, and compared their goodness of fit in terms of DIC. This is represented in figure S5:

***Figure S5. Model comparison.*** *We compared the main model, which contains alpha and beta parameters fitted at the group level, to four additional models. Goodness of fit was measured using the Deviance Information Criterion (DIC) and the differences (delta) between the lowest-scoring (i.e., best) model and the other models are reported, with values closer to zero indicating better performance. We tested two reduced models, one with the two alpha parameters (but not beta) and one that contains only one alpha parameter. Given that the parameters were fitted at the group level, we also fitted these models in a hierarchical fashion, such that both group-level and individual-level parameters were estimated. Results of model comparison are reported separately for each pattern. The DIC for the model with alpha and beta parameters (dic_ab) was the best for the high-drift pattern, while the model with two alpha parameters (dic_a) was the best for the other three patterns (stable, high-noise, and high-change-point-probability). Given that the main model includes the alphas-only model (i.e., their fit is the same when beta = 0), these results indicate that the main model can capture performance on all patterns, but the alphas-only model fails when the drift (i.e., velocity) is high. For this reason, we decided to use the main model which includes both alphas and beta parameters. (dic_abi = model with alphas, beta, and fitted at the individual level, dic_ai = model with alphas, no beta, and fitted at the individual level, dic_oa = model with only one alpha, and no beta parameter).*

It must be noted that on instances in which the model containing only alphas is best, the full model containing both alphas and beta performs equally well, with values of beta that approximate zero. In other words, the full model collapses to the reduced model in some situations, but not when beta is needed (see also response to the point 1 raised by reviewer #1).

Notably, the delta-rule model is focused on learning rather than predicting exploration behaviors. Therefore, even if the best model does not include parameters at the individual level, this does not go against our expectations about individual differences in *exploration*. It is the logistic models that are focused on predicting exploratory behaviors. In the logistic models, we do find individual differences as a function of autistic traits.

b. I struggled to understand some aspects of the modelling, and think that the description of the modelling details and approach could be clearer. For example, it is not immediately clear how many parameters are being fitted, which are participant specific or which, if any, are specific to the four different animals (contexts) introduced in the task.

We apologize if these descriptions were not clear previously. Thanks to the reviewer’s suggestion, we now have restructured and extended the explanation of the different parameters in the text, which led to major changes throughout Section 2.5 and to Figure 3. For each parameter, we now mention whether they are specific to each animal or to each trial. We also stress that no parameters were specific to each individual, as they were fitted at the group level. This is now also supported by model comparison (Figure S5), which shows that models accounting for individual differences have a worse fit.

c. To help the above point, the authors could draw more explicit links between the model details in the Methods section and Figure 3. The legend for Figure 3 could offer more explanation of the expressions on the right hand side of the figure

In the text, we have added explicit references to the plated structure of the model as displayed in Figure 3. In addition, we have added a more extensive explanation of the expressions in the caption of Figure 3.

d. Finally, whilst the paper clearly has a computational focus, reporting and plotting some model agnostic results may help the readers understanding. For instance, a demonstration of the effects of the different location trajectories (e.g. high drift, high changepoint probability etc) on p(leave) may be useful. It would also be reassuring to see individual datapoints for the key model agnostic results plotted.

We understand that the reviewer would want to see more model agnostic results. However, specifically plotting how the different patterns impact p(leave) might be misleading for two reasons. First, we introduced different patterns just to generate more variability in the task (and hence in the errors and learning progress that participants can make), but we are not interested in comparing the impact of the actual patterns on p(leave). Additional plots on this point might suggest that the patterns per se were subject of the investigation. Moreover, the trial-by-trial level of detail, which is so crucial in this analysis, would be lost, and we could only conclude whether one pattern is more likely than another to be abandoned by subjects. At the same time, we agree that it would be useful to show raw data more. For this reason, we have introduced Figure 2B, where the raw data of one example subject are plotted, together with the actual locations of the animals and the model fitted to the participant’s responses. In addition, we have added supplementary figure S2, which shows for each pattern in each environment the actual locations of the animals together with the mean and standard deviation of the responses of all participants:

***Figure S2. Participants’ predictions.*** *The mean (red dots) and standard deviation (red lines) of the participants’ predictions across every patter of every environment are reported. The actual location of the animals is depicted in blue. (pattern 1 = high drift, pattern 2 = high change-point probability, pattern 3 = high noise, pattern 4 = stable).*

We hope that these changes contribute to making the paper more transparent, starting from the raw data continuing across all analysis steps.

2. Trait measures: In its current form, the manuscript focuses on individual differences, which I consider an important step in understanding behaviour. However, I would like a few points to be clarified, or the focus of the paper to be adjusted to accommodate some concerns

a. Most of the significant results in the manuscript rest on “other” (parent) reports of autistic traits, rather than people’s self-report. Firstly, this is not clear from the abstract or title, and seems to be an important omission. I am not aware of any literature validating or advocating for the use of other people’s reports of autistic traits in adults, perhaps the authors can cite some papers supporting the reliability of this approach? Additionally, could the authors provide some motivation behind asking a parental figure to complete the ‘other’ reports, given the participants in this study are all adults. How should the reader interpret the findings where ‘other’ reported autistic traits do not align with the findings of ‘self’ reported autistic traits? Was there a specific hypothesis about other vs self-report measures? If so a direct statistical comparison of these measures should be included.

We have now added in the abstract that the effect we report regards other-reports. Moreover, we have added to the methods section the reason why we decided to prioritize other-reports (p. 7):

*“In the analyses, we primarily focused on other-reports collected from a parental figure. This decision was made based on the fact that individuals who have stronger autistic traits may also have more difficulty reflecting and reporting on their behavioral and emotional patterns or they may mask their patterns, which may result in more unreliable self-reports (Pearl et al., 2016). Indeed, previous research has found discrepancies between self- and other-reports (Lerner et al., 2012). For this reason, we expected parental reports to be more reliable. However, we always present results both for parental-reports and self-reports.”*

b. We would ask the authors to report more information about the ASBQ scores in this sample, for instance mean scores and ranges for the total score and subscales. It would also be helpful to see the relationship between self-report and other-report scores within participants.

We added the descriptive statistics for the ASBQ scores, as well as the correlation between self- and other-reports, to the supplementary figure S1:

***Figure S1. The preprocessing pipeline.*** *From the initial number of participants who were tested, seven were excluded because they did not follow the instructions. Of the remaining 70 participants, data from 31% percent of the settings were discarded because of boredom reports. All participants filled in the ASBQ self-reports, and 42 participants also had the other-reports filled in. The descriptive statistics and correlations between reports follow.*

c. The authors rightly seek to take the focus away from diagnosis and avoid groups, but then in the results binarize autistic traits into high or low. Aside from the problem of dichotomizing continuous variables (see Altman & Royston 2006 BMJ), could the authors explain why they chose to dichotomize (a movement back towards groups) in order to understand individual differences?

While in the analysis of leave-stay decisions we keep autistic traits as a continuous measure, we do binarize them when analyzing exploratory decisions. We were constrained in this decision by the R package mlogit that we used to analyze multinomial data (i.e., the decision of which character to choose among the three available characters) which does not allow to introduce interactions between variables that change trial by trial (e.g., learning progress and expected prediction error) and variables that change across subjects (e.g., autistic traits). As we report in the results section, we had to split the data and carry out two separate sets of analyses. We now state this more clearly in the results section:

*Multinomial logistic regression does not allow trial-by-trial variables (e.g., learning progress and expected prediction error) to interact with between-subject variables (e.g., ASBQ scores). Hence, to investigate differences across autistic traits, we did a mean split of each trait and ran two independent models for low and high scores.*

Moreover, we mention this as a limitation in the discussion:

*Finally, when analyzing the exploratory decisions, we were constrained by our analytic tools to split the sample in two groups and look at each subgroup’s results independently. This resulted in a loss of granularity in the analysis of exploratory decisions compared to our analysis of leave-stay decisions. Future studies should develop analytic methods to examine traits as continuous even in multinomial models.*

3. Framing of the paper: In its current form, the manuscript has many interesting threads, but readability and understanding may be improved if the number of threads was reduced

a. For instance, upon reading the whole paper, it is not clear how curiosity fits into the story with insistence on sameness? Could the authors either make this clearer, or make this thread slightly less apparent throughout the manuscript?

We thank the reviewer for pointing out that the connection between the different aspects of the paper can be improved. We tackled this issue in two ways. First, we mention the concepts of *curiosity* and *drives* more consistently across the paper. While it was mentioned explicitly only at the beginning of the introduction before, it now appears in the abstract and throughout introduction and discussion. We hope that this will make the link between uncertainty (e.g., where will the animal appear?) and the motive to resolve that uncertainty (i.e., curiosity) clearer. Second, we relate uncertainty intolerance/need for predictability more explicitly to insistence on sameness in the introduction:

*Given that the need for predictability might be the reason why some people entertain repetitive behaviors and have specialized interests, the primary focus of our analyses was on the subscale of insistence on sameness, which assesses these aspects (Caldwell-Harris & Jordan, 2014; Supekar et al., 2021).*

b. Additionally, in the methods, a factor of location trajectory (i.e. high drift, high noise etc) is proposed, but this does not really feature in the first two sections of the results, and is only mentioned briefly in the discussion. It may be the case that the output of the computational modelling includes this factor, but could the authors please clarify or expand on the role of this factor.

We introduced the different learning trajectories (i.e., patterns) to generate more variability in the task (and hence in the errors and learning progress that participants can make), but we were not interested in whether different patterns impact leave-stay or exploratory decisions as such. These factors appear only when assessing the reduction of prediction error over trials, because the patterns were independent of each other, also the resulting learning must be assessed separately. We now clarified this issue in the methods section:

*It should be noted that these patterns were introduced to generate variability in the task, and hence in the prediction errors and learning progress that participants would make. However, they were not the main focus of our analyses, which was when and what participants decided to explore.*

We also have the following minor notes:

1. We note a typo after the equation in Section 3.1 “where the autistic..”, where should be omitted.

2. Greater explanation of the error bars in Figure 4 and 5 would be helpful, given the complexity of interpreting high level interactions

3. It is not fully clear what constitutes a trial – perhaps Figure 1 could be reworked to indicate a full trial sequence in order along a row.

4. The authors refer to the model used as a hierarchical reinforcement learning model, which we think is a slight misuse of reinforcement, as there are no rewards in the task. We think a hierarchical learning model/hierarchical delta rule model may be more appropriate.

We changed the model name to hierarchical delta-rule model. All other typos and minor changes have been implemented.
