# Supplementary material for: Autistic traits foster effective curiosity-driven exploration
Source: PLoS Comput Biol. 2024 Oct 31;20(10):e1012453. doi: 10.1371/journal.pcbi.1012453 (PMC11527316; doi:10.1371/journal.pcbi.1012453)
Supplement: Attachment — Submitted filename: Response_reviewers.docx [file pcbi.1012453.s004.docx]

Reviewer's Responses to Questions

**Comments to the Authors:
Please note here if the review is uploaded as an attachment.**

Reviewer #1: I appreciate the authors’ effort in addressing all the points raised in the review. I just have one follow-up to the first major comment as below:

Comment #1:
Thank you for running this additional model fitting. It is now more clear which component of the hierarchical model becomes more relevant depending on the type of environment.
Yet, I’d still like to get some more clarifications on why this particular group-level fitting scheme was adopted, especially regarding whether group-fitting is appropriate for inferring individual variability. (This point was not addressed in the response letter.) I see that the group-level fit (dic_a or dic_ab) is overall better than the individual-level fit (dic_ai or dic_abi), but isn’t this again a comparison at a group level by averaging across all subjects? More precisely, aren’t the DIC of dic_ai and dic_abi models in Figure S5 computed by taking average across individuals? What I am trying to get at is whether there is any variability in terms of which model best account for each participant’s behavior (dic_ai vs. dic_abi).

Although the comparison is done at the group level, we believe that it takes well into account individual differences as well. For example, if different individuals were better fitted by different models, the model with both alpha and beta learning rates *and* hierarchical fitting should perform best. This is because it could have any of the parameters (e.g., beta) converge towards zero for some participants but not others. However, the hierarchical model performs worse, thus suggesting that it is not parsimonious to assume that different models might best account for the performance of different individuals. We would still like to stress that, although the delta-rule model itself is not capturing individual differences in parameters estimates, we were interested in differences in exploration rather than learning, and this is why we focus our analyses on leave-stay decisions and exploratory decisions, where individual differences do indeed emerge.

Also, Figure 3 caption states “All parameters were estimated across all individuals, but separately for each animal a,” but the following methods section (below Eq. 8) mentions only the learning rates. These two can be made consistent.

We thank the reviewer for spotting the mistake. We fit learning rate parameters (alphas and beta), and one additional parameter (z). Hence, referring to all parameters as “learning rates” is not entirely correct. This has now been corrected in the text after Eq. 8.

Comment #2.
Thank you for adding the additional panel for the example session. It is now much more clear and intuitive.

Comment #3:
Thank you for running this extra analysis on RT. The results are interesting but I agree that it is better kept out of the manuscript.

Typo - caption for Figure S2: every “patter” of every environment

The typo has been corrected.

Reviewer #2: The authors have addressed my prior concerns.

Reviewer #3: The authors have for the most part addressed my concerns satisfactorily and I am now happy to recommend publication.
